# *Additional sex combs-like 1* variants in an acute myeloid leukaemia and a general elderly cohort in central South Africa

**DOI:** 10.4102/ajlm.v15i1.3061

**Published:** 2026-08-26

**Authors:** Melissa V. Bergman, Jean F. Kloppers, Phillip A. Bester, Anne-Cecilia van Marle

**Affiliations:** 1 Department of Haematology and Cell Biology, Faculty of Health Sciences, University of the Free State, Bloemfontein, South Africa; 2 National Health Laboratory Service, Bloemfontein, South Africa; 3 Division of Virology, Faculty of Health Sciences, University of the Free State, Bloemfontein, South Africa

**Keywords:** *additional sex combs-like 1*, *ASXL1*, acute myeloid leukaemia, clonal haematopoiesis of indeterminate potential, epigenetics, adverse prognostic features

## Abstract

**Background:**

In patients with acute myeloid leukaemia (AML), pathogenic variants in the *additional sex combs-like 1* (*ASXL1*) gene confer poor prognosis and are frequently involved in clonal haematopoiesis of indeterminate potential, with increasing prevalence with advancing age.

**Objective:**

We determined the prevalence of *ASXL1* variants in an AML cohort and general elderly population in central South Africa.

**Methods:**

The study included 40 participants with de novo AML and 100 elderly participants (≥ 65 years). Polymerase chain reactions and Oxford nanopore sequencing of *ASXL1* exon 12 were performed on all samples between 01 October 2023 and 30 September 2024.

**Results:**

Of the 40 AML participants, 25 (62.5%) were women, with a median age of 42 years. *ASXL1* mutations were detected in 5%. In total, 248 *ASXL1* variants were detected in 68 of the 100 elderly participants aged 65–88 years (mean 71 years). Variants in the coding regions included synonymous (58.4%) and missense (41.6%) variants. Benign variants were detected in 29% of the elderly participants, while variants of uncertain significance were present in 2%, each with a variant allele frequency of ≥ 20%. No pathogenic or likely pathogenic variants were identified.

**Conclusion:**

The prevalence of *ASXL1* exon 12 variants in our AML cohort was consistent with international data. Variants in the elderly population were very prevalent; however, with no actionable variants capable of driving clonal expansion, clonal haematopoiesis of indeterminate potential was not detected. The variants of uncertain significance at allele frequencies of ≥ 2% likely suggest a clonal haematopoietic process.

**What this study adds:**

Routine *ASXL1* testing in all AML patients is not recommended in our resource-limited setting. Testing should be individualised according to its potential clinical impact, using high-throughput sequencing with clinically relevant variant classification.

## Introduction

Acute myeloid leukaemia (AML) is a heterogeneous disease characterised by arrested myeloid maturation, leading to the accumulation of myeloid blasts in the bone marrow, peripheral blood and other tissue.^1^ Acute myeloid leukaemia is the most common acute leukaemia in adults, with a male predominance. In the United States, the median age at diagnosis is 69 years.^2,3^ However, previous South African studies have reported a significantly younger median age at 42 years.^2,3,4^ In the United States, the reported incidence is 4.2 cases per 100 000 population per year. The estimated median overall survival (OS) is 8.5 months, with the worst median OS (2.67 months) when diagnosed at age ≥ 65 years.^2^ Limited data are currently available regarding the epidemiology of AML in South Africa.^4^

Recurrent chromosomal aberrations detected by conventional cytogenetics and other methods, such as fluorescence in situ hybridisation or next-generation sequencing, form the cornerstone of prognostication in AML, assigning patients to one of three main risk groups: favourable, intermediate, or adverse (Table 1).^5^ Patients with cytogenetically normal AML, approximately 45% of adult cases, were previously categorised in the intermediate-risk group. However, several subsequent studies have identified additional molecular abnormalities that modify this risk stratification.^5,6,7^

**TABLE 1:** Adapted 2022 European Leukemia Net risk classification of acute myeloid leukaemia according to genetics, Bloemfontein, South Africa, 01 October 2023 – 30 September 2024.

Risk category	Genetic abnormality
Favourable	• t(8;21)(q22;q22.1)/*RUNX1::RUNX1T1* • inv(16)(p13.1q22) or t(16;16)(p13.1;q22)/ *CBFB::MYH11* • Mutated *NPM1*, without *FLT3-ITD* • bZIP in-frame mutated *CEBPA*
Intermediate	• Mutated *NPM1*, with *FLT3*-ITD • Wild-type *NPM1* with *FLT3*-ITD (without adverse-risk genetic lesions) • t(9;11)(p21.3;q23.3)/*MLLT3::KMT2A* • Cytogenetic and/or molecular abnormalities not classified as favourable or adverse
Adverse	• t(6;9)(p23.3;q34.1)/*DEK::NUP214* • t(v;11q23.3)/*KMT2A* rearranged • t(9;22)(q34.1;q11.2)/*BCR::ABL1* • t(8;16)(p11.2;p13.3)/*KAT6A::CREBBP* • inv(3)(q21.3q26.2) or t(3;3)(q21.3;q26.2)/ *GATA2, MECOM(EVI1)* • t(3q26.2;v)/*MECOM*(*EVI1*)-rearranged • -5 or del(5q); -7; -17/abn(17p) • Complex karyotype, monosomal karyotype • Mutated *ASXL1, BCOR, EZH2, RUNX1, SF3B1, SRSF2, STAG2, U2AF1*, and/or *ZRSR2* • Mutated *TP53*

*Source*: Adapted from: Döhner H, Wei AH, Appelbaum FR, et al. Diagnosis and management of AML in adults: 2022 recommendations from an international expert panel on behalf of the ELN. Blood. 2022;140(12):1345–1377. https://doi.org/10.1182/blood.2022016867^11^

BCOR, BCL6 corepressor; EZH2, enhancer of zeste homolog 2; RUNX1, runt-related transcription factor 1; SF3B1, splicing factor 3b subunit 1; SRSF2, serine and arginine rich splicing factor 2; STAG2, stromal antigen 2; U2AF1, U2 small nuclear RNA auxiliary factor 1; ZRSR2, zinc finger CCCH-type, RNA binding motif and serine/arginine rich 2; RUNX1T1, RUNX1 translocation partner 1 (formerly ETO); CBFB, core-binding factor beta sub-unit; MYH11, myosin heavy chain 11; NPM1, nucleophosmin 1; FLT3-ITD, FMS-like tyrosine kinase 3, internal tandem duplication; CEBPA, CCAAT enhancer binding protein alpha; KMT2A, lysine methyltransferase 2A (formerly MLL); MLLT3, MLLT3 super elongation complex subunit (formerly AF9); DEK, DEK proto-oncogene; NUP214, nucleoporin 214; BCR, breakpoint cluster region; ABL1, ABL proto-oncogene 1, non-receptor tyrosine kinase; KAT6A, lysine acetyltransferase 6A; CREBBP, CREB binding protein; GATA2, GATA binding protein 2; MECOM (EVI1), MDS1 and EVI1 complex locus (ecotropic viral integration site 1); TP53, tumor protein p53.

Mutations involving epigenetic regulators, considered key events in AML leukaemogenesis, account for some of the most frequent recurrent molecular abnormalities in adult cytogenetically normal AML. These include mutations in genes encoding *DNA methyltransferase 3 alpha* (*DNMT3A*) (30% – 37%), *isocitrate dehydrogenase 1* and *isocitrate dehydrogenase 2* (*IDH1* and *IDH2*) (25% – 30%), *additional sex combs-like 1* (*ASXL1*) (5% – 12%) and *Tet methylcytosine dioxygenase 2* (*TET2*) (9% – 23%).^6,8^ The discovery of these mutations has significantly contributed to our understanding of the pathogenesis of AML and proved to be powerful prognostic determinants.^9^

In 2022, the 5th edition of the World Health Organization (WHO) Classification of Haematolymphoid Tumours,^10^ as well as the new International Consensus Classification by the European Leukemia Net,^11^ published updated AML classification systems. Both systems emphasise the importance of molecular analysis in AML workup. Although these two classification systems differ in certain diagnostic criteria and AML subtypes, both systems include a category with ‘myelodysplasia-related’ gene mutations, which confer poor prognosis and are classified in the adverse-risk category (Table 1). One of the most frequently mutated genes in myeloid malignancies, the *ASXL1* gene, is included in this category.^11^

*Additional sex combs-like 1* is located on chromosome 20q11, consists of 12 exons and encodes a 1541 amino acid protein.^12^ In humans, ASXL proteins function as epigenetic scaffolds that assemble transcription factors and histone modification complexes, resulting in transcriptional activation or repression of genes involved in cell differentiation and proliferation.^12,13^ *Additional sex combs-like 1* mutations contribute to the malignant transformation of myeloid cells due to abnormal histone modification through a gain-of-function effect of the truncated protein.^12^ Somatic mutations of *ASXL1* have been identified in patients with all types of myeloid malignancies and almost always involve exon 12 of the gene.^14,15,16^ The prevalence of *ASXL1* mutations in AML varies from 6.5% in de novo AML to approximately 30% in AML secondary to an antecedent haematologic neoplasm or a history of previous exposure to chemotherapy or radiotherapy.^15^

*Additional sex combs-like 1* mutations are early events in AML and are associated with a poor prognosis owing to the aggressive nature of the disease and resistance to treatment.^11,17^ Various studies reported that patients with the *ASXL1* mutation manifested with a worse complete remission rate, significantly shorter OS and lower event-free survival compared to patients without the mutation.^7,18,19,20^ The *ASXL1* mutation is considered an independent adverse prognostic factor for survival in AML patients.^21,22^

Clonal haematopoiesis refers to a subpopulation of myeloid cells that share a somatic mutation, distinguishing them from unaffected haematopoietic and non-haematopoietic cells.^23^ Early events initiate alterations in haematopoietic stem cells, generating ‘pre-leukaemic’ cells with a clonal advantage.^14,24^ However, without other mutations or epigenetic changes, these pre-leukaemic haematopoietic stem cells do not cause malignant transformation of downstream progenitor cells and may never progress to clinical disease.^24,25^ Clonal haematopoiesis of indeterminate potential (CHIP) is defined as a pathogenic or likely pathogenic variant with a variant allele frequency of ≥ 2% of the somatic mutation of a leukaemia-associated gene in the absence of cytopenias and a WHO-defined haematological malignancy.^23^ The most commonly mutated genes associated with CHIP are *DNMT3A, TET2* and *ASXL1*, collectively called DTA. These mutations have been reported in up to 10% of seemingly healthy adults aged ≥ 65 years.^9,26,27,28^

The prevalence of *ASXL1* mutations is unknown in both the central South African general elderly population and the AML population. This study aimed to determine the prevalence of *ASXL1* mutations in an AML cohort and a general elderly population in central South Africa using Oxford nanopore sequencing, a high-throughput sequencing method. This platform enables rapid, high-throughput genomic profiling through the parallel sequencing of millions of DNA fragments, fundamentally advancing precision medicine compared to traditional Sanger sequencing approaches.^29^ Oxford nanopore sequencing has a high-accuracy base-calling module, with an estimated F1 score of 99.5% at 20× coverage.^30^

We hypothesised that if *ASXL1* mutations are prevalent in our general elderly population, their presence in AML patients of our region may merely reflect background clonal haematopoiesis and not necessarily confer adverse prognosis. In support of this hypothesis, we attempted to evaluate *ASXL1*-mutational status in our AML patients in the context of clinical presentation and outcomes.

## Methods

### Ethical considerations

Ethics approval for the study was obtained from the Health Sciences Research Ethics Committee (HSREC) of the University of the Free State (approval number: UFS-HSD2023/0961/2609), and the Free State Provincial Department of Health (approval number FS_202309_006). The study complied with the South African *Protection of Personal Information Act (POPIA) of 2013*. Participants in the AML cohort were recruited as part of a previous study UFS-HSD 2020/1327/2710) and signed a genetic informed consent form, allowing future AML research to be conducted on their blood samples. The AML study samples were pseudonymised to uphold confidentiality, and participant data were only accessible to the researchers. For the samples used in the general elderly population cohort, no informed consent was required, as these samples were de-identified by a third-party individual independent of this study. The researchers only received numbered blood samples and a list with corresponding study numbers, participant age and sex for each sample.

### Study population

A descriptive, observational study was conducted at the Universitas National Health Laboratory Service service laboratory in Bloemfontein, Free State province, South Africa. Data collection commenced on 01 October 2023 and was concluded on 30 September 2024. A total of 40 archived samples (designated P1–P40) of adult participants (≥ 18 years) previously diagnosed with de novo AML were included in this study. These samples were kept in a –20 °C freezer in the Tissue Typing laboratory within the Department of Haematology and Cell Biology. In addition, 100 (S1–S100) peripheral blood samples of patients representing a general elderly population of central South Africa were included. A third-party individual, independent of this study, screened all full blood count requests submitted to the Universitas Academic Hospital National Health Laboratory Service Mservice laboratory, which receives samples from a wide drainage area across the Free State, Northern Cape and North West provinces, including both tertiary and peripheral healthcare facilities, and randomly selected the ethylenediaminetetraacetic acid specimens of patients aged ≥ 65 years with normal full blood count parameters. This cohort was therefore not restricted to any specific ward, clinic, or clinical discipline, but rather reflects a heterogeneous population of older individuals accessing routine laboratory services within the catchment area. The de-identified samples were assigned study numbers in ascending order of collection.

### DNA isolation and quantification

Genomic DNA was isolated from 200 µL whole blood samples using the Quick-DNA™ Miniprep Kit (Zymo Research; Irvine, California, United States). The archived DNA samples from the AML participants were previously extracted using the same methodology and stored at –20 °C until analysis. DNA quantification was done using the SpectraMax^®^ QuickDrop™ Micro-Volume Spectrophotometer (Molecular Devices LLC; San Jose, California, United States) according to the manufacturer’s instructions.

### Polymerase chain reaction prior to sequencing

Published primers were used to amplify *ASXL1* exon 12 of all participant samples using long-range polymerase chain reaction (PCR). The PCR consisted of 5 µL of 5X Green GoTaq^®^ Flexi buffer (Promega; Madison, Wisconsin, United States), 1 µL MgCl_2_ (2.5 mM), 1 µL forward (5’-TCACACAGTCCCACCAGAAA-3’) and reverse primer (5’-TTAGGCAGGAGCACTCTTGC-3’) (100 nM) (Inqaba Biotec; Muckleneuk, Pretoria, South Africa), 0.5 µL of dNTPs (200 µM) (New England Biolabs; Ipswich, Massachusetts, United States), 0.25 µL of GoTaq^®^ G2 Flexi DNA Polymerase (5 U/µL) (Promega, United States), 1 µL of DNA (50 ng/µL), and 10.25 µL of nuclease-free water, to obtain a final reaction volume of 25 µL. The PCR cycling conditions included an initial denaturation at 95 °C for 5 min, 30 cycles of denaturation at 95 °C for 30 s, annealing at 55 °C for 30 s, elongation at 72 °C for 45 s, and a final elongation step of 72 °C for 7 min. Gel electrophoresis of PCR products was conducted at 120 V for 60 min on a 1% SeaKem LE Agarose gel (Lonza; Walkersville, Maryland, United States).

### High throughput sequencing analysis of *additional sex combs-like 1 exon 12*

Oxford Nanopore Sequencing (Oxford Nanopore Technologies [ONT]; Oxford, United Kingdom) was used for sequencing the *ASXL1 exon 12* for every sample.

Amplicons for each sample were barcoded using the Ligation sequencing amplicons – Native Barcoding Kit 96 V14 (SQK-NBD114.96) (ONT, United Kingdom). The barcoded amplicon was loaded onto a Flongle flow cell (R10.4.1) on the MinION device (ONT, United Kingdom). Base calling was done using Guppy (v6.5.7) (ONT, United Kingdom) with a minimum quality score of Q10. The resulting demultiplexed FASTQ files were mapped against chromosome 20 of the human reference genome, GRCh38, using Minimap2 (ONT, United Kingdom). Consensus sequences were created using samtools consensus (v1.22.1, 2005, Samtools Development Team, Wellcome Sanger Institute; Cambridge, United Kingdom) with a minimum depth of 40 times coverage. These sequences were then polished using medaka (v1.12.1, 2024, ONT PLC; Oxford, United Kingdom). The BAM files from the initial mapping with Minimap2 were subjected to variant calling using VarScan (v2.4.6, 2022, The Genome Institute, Washington University School of Medicine; St. Louis, Missouri, United States). The resulting VCF files were uploaded to the Ensembl Variant Effect Predictor tool (European Bioinformatics Institute and Wellcome Sanger Institute; Hinxton, Cambridgeshire, United Kingdom) for sequence alignment, functional annotation, predicted impact on protein function, and genomic context.

### Data analysis

Sequences were aligned to the NCBI *ASXL1* reference sequence (NC_000017.11, NM_000546.6, hg38) (available at https://ncbi.nlm.nih.gov) using the EMBOSS Needle online sequence alignment tool (available at https://www.ebi.ac.uk/jdispatcher/psa/emboss_needle).^31^ Variant nomenclature was assigned according to the Human Genome Variation Society guidelines (available at https://hgvs-nomenclature.org/stable/hvnc/).^32^ Variants were characterised according to mutation effect, location and position (coding and protein sequence position). Pathogenicity of all variants were described using prediction software, including VarSome (available at https://varsome.com/),^33^ ClinVar (available at https://www.ncbi.nlm.nih.gov/clinvar),^34^ and the Catalogue of Somatic Mutations in Cancer (available at https://cancer.sanger.ac.uk).^35^

### Evaluating the prevalence and clinical association of *additional sex combs-like 1*

The prevalence of *ASXL1* variants (benign and pathogenic) for the AML cohort and the general elderly population was determined and expressed as a percentage of the total number of participants per cohort. For the AML cohort, limited demographic and clinical data were collected as part of the original study. Clinical data included information regarding the diagnosis, treatment received, treatment response, early mortality and disease relapse. This information was used to evaluate the association between *ASXL1* mutational status and participant clinical characteristics.^8^

## Results

### The acute myeloid leukaemia cohort

Twenty-five (62.5%) of the 40 participants were women. The median age of the cohort was 42 years old (interquartile range [IQR] 18–70 years old). The AML subtypes were classified according to the 5th edition of the WHO Classification of Haematolymphoid Tumours^10^ as either AML with defining genetic abnormalities or AML defined by differentiation (Table 2). Genetic testing, however, was limited to conventional karyotyping, fluorescence in situ hybridisation for t(8;21)(q22;22.1)/*RUNX1::RUNX1T1*, t(16;16)(p13.1;q22)/inv(16)(613.1q22)/*CBFB::MYH11*, t(9;22)(q34.1;q11.2)/*BCR::ABL1*, 11.q23.3/*KMT2A* rearrangement, and PCR for *FLT3*-ITD and *NPM1* mutations. Myeloid next-generation sequencing was only performed on selected patients to aid with clinician treatment decisions.

**TABLE 2:** The acute myeloid leukaemia cohort (*n* = 40) subtypes of acute myeloid leukaemia according to the 5th edition of the World Health Organization Classification of Haematolymphoid Tumours classification.

Category	Subtype	*n*
	Additional cytogenetic and/or molecular abnormalities	
AML with defining genetic abnormalities	***RUNX1::RUNX1T1***	12
	*ASXL1*	1
	Loss of chromosome Y	4
	Loss of chromosome X	1
	Additional chromosome 8	1
	*NPM1* mutation	1
	17p13.1 deletion of *TP53*	1
	Complex karyotype	1
	t(7;9)	1
	t(4;21;8)(q21;q22;q22	1
	***CBFB::MYH11***	4
	*FLT3*-ITD	1
	Trisomy 8	1
	17p13.1 deletion *TP53*	1
	***BCR::ABL1***	1
	der(5)t(1;5)(q?25q?35)	
	***KMT2A* rearrangement**	1
	***MECOM* rearrangement**	1
	Complex karyotype	
	***Myelodysplasia-related* AML**	1
	del 5q31 and 5q33	
	Monosomy 7; deletion of 7q22q31	
AML defined by differentiation	**AML with minimal differentiation**	4
	deletion of 21q22.1 – *RUNX1*	1
	*ETV6* gene mutation	1
	17p13.1 deletion of *TP53*	1
	**AML without maturation**	3
	*FLT3*-ITD and *NPM1*	1
	**AML with maturation**	4
	Hyperdiploidy *RUNX1T1* (8q21) and *RUNX1* (21q22)	1
	*ASXL1* mutation	1
	Complex karyotype	1
	**Acute myelomonocytic leukaemia**	6
	*FLT3*-ITD mutation	1
	*RUNX1* and *PTPN11*	1
	Complex karyotype	1
	**Acute monocytic leukaemia**	3
	*FLT3*-ITD and *NPM1*	1

*Source*: Khoury JD, Solary E, Abla O, et al. The 5th edition of the World Health Organization classification of haematolymphoid tumours: Myeloid and histiocytic/dendritic neoplasms. Leukemia. 2022;36(7):1703–1719. https://doi.org/10.1038/s41375-022-01613-1^10^

AML, acute myeloid leukaemia; *FLT3*-ITD, *FMS-like tyrosine kinase 3* – internal tandem duplication; ETV6, ETS variant transcription factor 6; PTPN11, protein tyrosine phosphatase non-receptor type 11; NPM1, nucleophosmin 1.

The most common WHO subtype diagnosed was AML with defining genetic abnormalities, with the highest frequencies reported in the core binding factor AML *RUNX1::RUNX1T1* at 30% (*n* = 12/40). Lower frequencies were detected in *CBFB::MYH11* at 10% (*n* = 4/40). *ASXL1* mutations were only detected in two (5%) of the 40 participants. No benign variants or variants of uncertain significance were detected in the AML cohort. The low prevalence of *ASXL1* mutations in the AML cohort (*n* = 2/40; 5%) limited comparison between the *ASXL1*-positive (*ASXL1*pos) and *ASXL1*-negative (*ASXL1*neg) AML participants.

The demographic and limited clinical data of the two *ASXL1*pos participants, as well as the mutation characteristics and clinical significance, are summarised in Table 3. Only one of the *ASXL1*pos participants (P7) received standard induction chemotherapy (3 + 7: short infusions of daunorubicin for the first three days + continuous infusion of cytarabine for 7 days), and both participants demised within 3 months of diagnosis (P4: 5 days; P7: 64 days).

**TABLE 3:** Demographic, clinical and mutation characteristics of the *ASXL1*-positive patients with acute myeloid leukaemia, Bloemfontein, South Africa, 01 October 2023 – 30 September 2024.

Identifier	Age (years)	Sex	AML subtype	Treatment	Outcome	*ASXL1* mutation	Mutational variant	VAF (%)	Clinical significance
P4	61	M	AML with maturation	Did not receive 3 + 7†	Demised (5 days)	NM_015338.6*(ASXL1)*:c.2362G>T:p.Glu788‡	Nonsense mutation	28.57	Pathogenic
P7	18	M	AML with *RUNX1::RUNX1T1*	Induction therapy 3 + 7† Consolidation	Demised (64 days)	NM_015338.6(*ASXL1*):c.1900_1922delAGAGAGGCGGCCACCACTGCCAT:p.Glu635fs§	Frameshift mutation	48.10	Pathogenic

AML, acute myeloid leukaemia; M, male; VAF, variant allele frequency; *ASXL1, additional sex combs-like 1*.

† , 3 + 7: short infusions of daunorubicin for 3 days + continuous infusion of cytarabine for 7 days;

‡ , Catalogue of Somatic Mutations in Cancer database (available at https://cancer.sanger.ac.uk);^35^

§ , VarSome (available at https://varsome.com/)^33^ and ClinVar (available at https://www.ncbi.nlm.nih.gov/clinvar).^34^

At the time of writing the manuscript, eight of the *ASXL1*neg cohort were still alive, eight participants were lost to follow-up (presumed deceased, because of abrupt termination of clinical documentation and laboratory results), one participant refused hospital treatment, and 23 participants were confirmed deceased (inclusive of the *ASXL1*pos cases). The median survival for the deceased *ASXL1*neg participants was 20 days (IQR 2–664 days). When comparing the OS of the two *ASXL1*pos participants to that of *ASXL1*neg participants of the same age, the younger cohort (*n* = 12/40; age 18–30 years) had a median survival of 228.5 days (IQR 1–1385 days), while the older cohort (*n* = 8/40; age 65–76 years) had a median survival of 11 days (IQR 5–40 days).

### The elderly population

**FIGURE 1:**
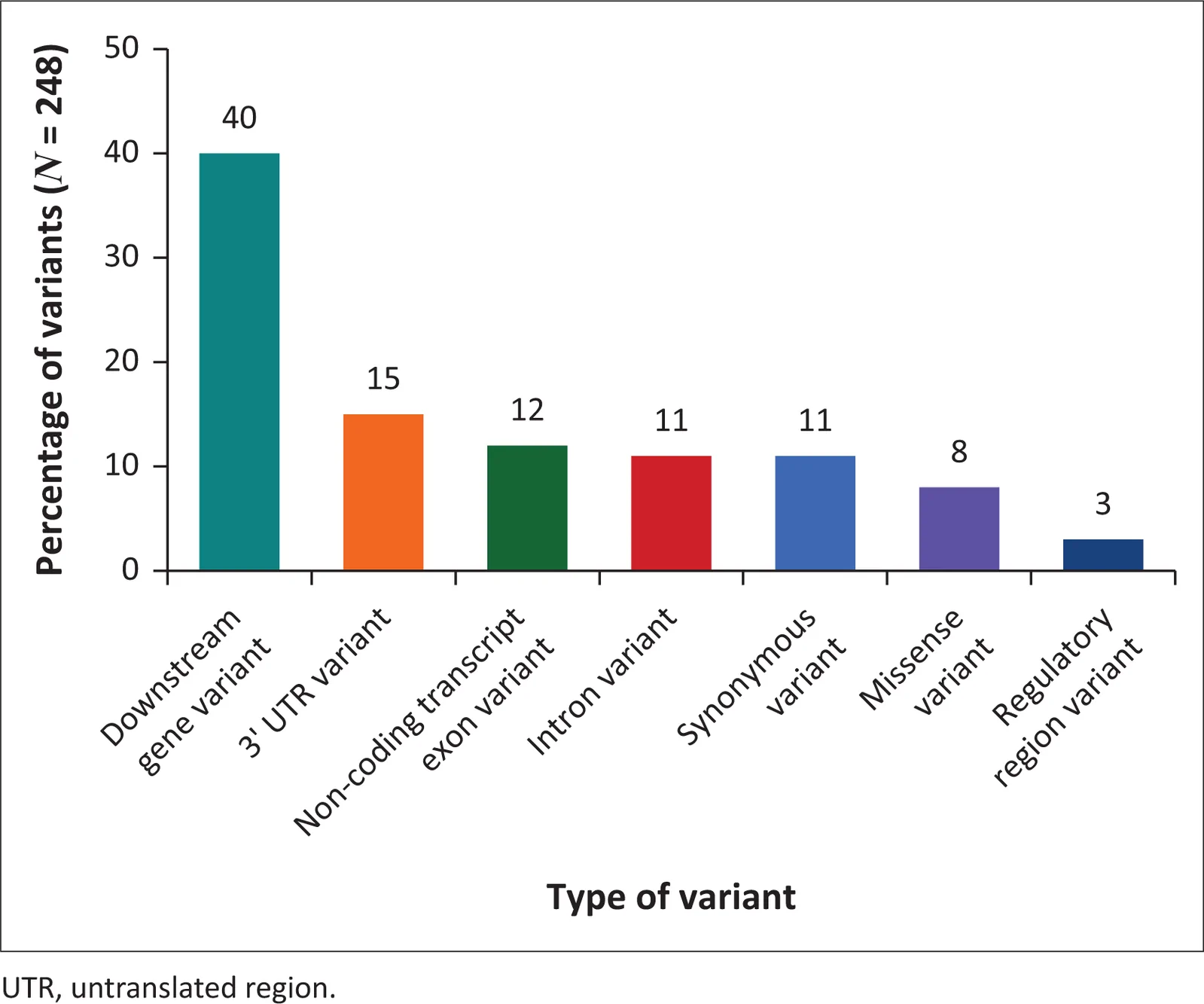
Distribution of variants in the coding (*n* = 226) and non-coding (*n* = 22) regions of the elderly population (compiled from Ensembl-generated results), Universitas NHLS, Bloemfontein, South Africa, 01 October 2023 – 30 September 2024.

Sixty-three of the 100 participants were women. The cohort had an age range of 65–88 years (mean 71 years), with 43 participants aged 65–69 years, 46 aged 70–79 years, and 11 aged 80–88 years. A total of 248 *ASXL1* variants were detected in the coding (*n* = 226/248) and non-coding regions (*n* = 22/248) (Figure 1).

Variants in the coding regions included synonymous (*n* = 132/226; 58.4%) and missense (*n* = 94/226; 41.6%) variants and were detected in 68 of the 100 participants. Participants either had a missense variant only (*n* = 11/68; 16.2%), a combination of a missense and synonymous variants (*n* = 20/68; 29.4%), or a synonymous variant only (*n* = 37/68; 54.4%). Sixteen different missense variants were detected, of which 87.5% (*n* = 14/16) were benign and detected in 29% (*n* = 29/100) of the participants. Two different variants (*n* = 2/16; 12.5%) of uncertain significance were detected in 2% (*n* = 2/100) of the participants, with a variant allele frequency of 20% (*ASXL1*:c.3934C>A) and 46% (*ASXL1*:c.2113G>A), respectively. No pathogenic or likely pathogenic variants were identified as per the American College of Medical Genetics and Genomics and the Association for Molecular Pathology guidelines.^36^

Nineteen of the participants with missense variants (with or without synonymous variants) were women (*n* = 19/31; 61.3%). Missense variants were detected at 55.3% (*n* = 52/94) in 41.9% (*n* = 18/43) of the 65–69 year old age group, followed by 40.4% (*n* = 39/94) in 23.9% (*n* = 11/46) of the 70–79 year old group, and 4.3% (*n* = 4/94) in 18.2% (*n* = 2/11) of the 80–89 year old group.

All participants with missense variants (*n* = 31) harboured two or more variants, including six variants in 9.7% (*n* = 3/31) of the participants, four variants in 35.5% (*n* = 11/31) of the participants, and two variants in 54.8% (*n* = 17/31) of the participants. The two most prevalent variants, each accounting for 19.1% of all missense variants (*n* = 18/94), were *ASXL1*:c.3790C>T and *ASXL1*:c.3958C>T. The variants *ASXL1*:c.2330A>G, *ASXL1*:c.2498A>G, *ASXL1*:c.2236G>A and *ASXL1*:c.2068G>A were each detected at 11.7% (*n* = 11/94).

## Discussion

### Acute myeloid leukaemia population

Mutations involving exon 12 of the epigenetic regulator gene, *ASXL1*, contribute to the malignant transformation of myeloid cells and confer a poor prognosis associated with aggressive disease and resistance to treatment.^11,17^ The prevalence of *ASXL1* mutations in AML ranges from an average of 6.5% in de novo AML to almost five times higher in secondary AML.^15^ *Additional sex combs-like 1* exon 12 mutations were detected in 5% (*n* = 2/40) of our AML study cohort, which was consistent with data reported internationally (5% – 12%).^6,8^ A significantly younger median age at diagnosis of 42 years was reported in our AML cohort. Although not unusual in the South African context, it was notably lower than the internationally reported median age of 63–71 years at the time of AML diagnosis.^2,4,5^ *Additional sex combs-like 1* mutations are five times more prevalent in older patients (≥ 60 years); however, while rare in younger individuals, some cases have been reported.^20,37,38^

Both patients in our study with the *ASXL1* mutation were men; one (P4) was diagnosed at the age of 61 years, while the other (P7) was much younger at 18 years. One can deduce that older age was not the main determinant in the poor outcome of these two patients, both of whom demised within 3 months of presentation. However, when comparing the OS of two *ASXL1*pos participants to that of *ASXL1*neg participants of the same age, the younger cohort (age 18–30 years) had a median survival of 228.5 days, contrary to the cohort aged 65–76 years, with a median survival of 11 days. Therefore, at least in the younger AML cohort, *ASXL1* mutational status apparently contributed to worse OS. However, in a resource-constrained setting, several unmeasured confounding factors are likely to have influenced these outcomes. These include staff shortages limiting the capacity for close monitoring of critically ill patients, as well as restricted access to intensive care unit beds and isolation facilities. Such constraints are likely to contribute to a higher burden of infectious complications and early mortality.

In line with this assessment, the *ASXL1* variant detected in our young AML participant (P7) was a frameshift mutation (c.1900_1922del) that was classified as pathogenic. Previous studies that detected the same mutation also reported an inferior OS.^37,39^ Frameshift and nonsense variants are most frequently detected in AML, although missense mutations are not uncommon.^21,38^ At the time of writing this manuscript, no literature has been published on the nonsense variant (c.2362G>T) that was detected in AML participant P4; this variant, however, has been reported in AML on the Catalogue of Somatic Mutations in Cancer database: COSM 6927846.^35^ This variant was also absent in our general elderly population.

It is noteworthy that the most common WHO subtype diagnosed was AML with defining genetic abnormalities, *RUNX1::RUNX1T1* fusion that was detected in 30% of the AML cohort. Both core binding factor AML subtypes, *RUNX1::RUNX1T1* and *CBFB::MYH11*, were observed at higher frequencies in this study than that reported locally^4,40^ and internationally.^41^ These data emphasised the genetic differences in populations, both locally and internationally. Core binding factor AML is classified in the favourable risk category group, and is typically associated with a good response to induction chemotherapy and high complete remission in AML patients.^11,42^ *Additional sex combs-like 1* is often co-mutated in AML with *RUNX1::RUNX1T1* fusion and has not shown to alter the prognosis or adversely affect OS and event-free survival.^17,21,43^ Yet, our *ASXL1*pos AML participant (P7) with the *RUNX1::RUNX1T1* fusion demised 64 days after diagnosis. A possible confounder contributing to P7’s short survival could be the additional loss of chromosome Y.

The prognostic impact of loss of chromosome Y in AML with *RUNX1::RUNX1T1*, however, remains controversial, with some studies suggesting a favourable outcome, while others found it to be a poor prognostic indicator.^44,45^ Our observation seems to support the latter. Of note, loss of chromosome Y in ≥ 75% of metaphases is strongly associated with mutations in myeloid neoplasm-related genes, including *ASXL1, TET2* and *DNMT3A*.^46^

### The general elderly population

*Additional sex combs-like 1* is the third most commonly mutated driver gene reported in CHIP.^12^ The prevalence of CHIP is negligible during childhood and young adulthood but steadily increases with advancing age, and has been reported in up to 10% of seemingly healthy adults aged ≥ 65 years.^23,27,28,47,48^ *Additional sex combs-like 1* exon 12 variants were highly prevalent in our general elderly cohort at 68%, with missense (41.6%) and synonymous variants (58.4%) detected in the coding regions, and all of the participants harbouring two or more variants. According to the American College of Medical Genetics and Genomics and the Association for Molecular Pathology guidelines,^36^ none of these variants was classified as pathogenic or likely pathogenic. Benign variants having no clinical significance or association with disease were identified in 29% of the elderly participants. Furthermore, variants of uncertain significance were detected in two of the participants, with a variant allele frequency of 20% and 46%, respectively. Data related to both of these variants are limited, emphasising the need for future studies to determine their clinical significance. However, both variant allele frequencies provide strong evidence for a clonal haematopoietic process.

Jongen-Lavrencic et al.^49^ performed targeted next-generation sequencing at diagnosis and after induction chemotherapy on 482 patients newly diagnosed with AML. The study endpoints were 4-year cumulative incidence of relapse, OS, and relapse-free survival. The persistence of *DNMT3A, TET2* and *ASXL1* mutations during complete remission was not associated with an increased relapse rate or death, and did not appear to have prognostic value within the 4-year follow-up period. Instead, the cells harbouring *DNMT3A, TET2* and *ASXL1* mutations appeared to represent non-leukaemic clones with a selective clonal advantage over normal haematopoietic stem cells to repopulate the bone marrow.^49^ In accordance with this study, we hypothesised that *ASXL1* variants, particularly in elderly patients with AML, merely reflect the high prevalence of mutated *ASXL1* CHIP, and do not necessarily serve as an independent adverse prognostic indicator in AML. Unfortunately, the low prevalence of *ASXL1* variants in our AML cohort in general, and particularly in the elderly AML subgroup, could not provide evidence for our theory.

Considering the detrimental clinical implications of *ASXL1* variants in AML and the countless possible *ASXL1* variants in exon 12 alone, a pre-sequencing screening method, such as PCR-based high-resolution melting analysis, would seem practical in a resource-constrained diagnostic setting. Pre-sequencing screening methods for variant detection of leukaemic genes generally reduce the need to perform high-throughput DNA sequencing on all samples, thereby reducing cost, time and labour.^50^ However, with our high prevalence of *ASXL1* variants in the general elderly population, one could argue that pre-sequencing screening for *ASXL1* variants, particularly in elderly patients with AML, may result in unnecessary delays in risk stratification and treatment planning, as most of these patients would require subsequent DNA sequencing for confirmation and characterisation of the variants.

Bodian et al.^51^ performed whole exome sequencing on 158 cancer-susceptibility genes from a cohort of 681 healthy individuals. Based on their results, there was a 100% chance of identifying missense variants in cancer genes with whole exome sequencing, with an average of 68 variants per individual. The high number of variants of unknown significance underscored the need to characterise genetic variants to promote understanding of the clinical relevance and implementation.^51^ Therefore, with the high prevalence of non-pathogenic *ASXL1* variants in our general elderly cohort, the low prevalence of *ASXL1* variants in our AML cohort, and the overall poor clinical outcome of the AML participants, regardless of *ASXL1* mutational status or age, implementation of routine diagnostic testing – whether it be a screening of high-throughput sequencing method – may need to be tailored to the individual patient, particularly in a resource-limited setting.

### Limitations

In addition to the small sample size, only focusing on *ASXL1* variants detected in exon 12 could be regarded as a limitation of the study. The low prevalence of *ASXL1* variants in the AML cohort might be attributed to the lower median age at diagnosis, mostly including patients with AML with defining genetic abnormalities and excluding patients with secondary AML. The low *ASXL1* variant prevalence and limited clinical data available hampered true comparisons between the *ASXL1*pos and *ASXL1*neg AML participants. In addition, numerous confounding factors, such as comorbidities and disease- and treatment-related complications, may have contributed to or resulted in early mortality, irrespective of *ASXL1* mutational status. However, these variables were not investigated in our study. We only focused on the prevalence of *ASXL1* variants. However, future studies including the other *DNMT3A, TET2* and *ASXL1* CHIP variants, *TET2* and *DNMT3A*, and their association with cardiovascular disease, are recommended.

## Conclusion

The prevalence of *ASXL1* exon 12 variants in our AML cohort is consistent with data reported internationally and proved to be very prevalent in the general elderly population, with benign variants and variants of uncertain significance. However, no variants were classified as pathogenic or likely pathogenic in the elderly cohort and therefore, no CHIP was identified. We do not advocate routine testing for *ASXL1* variants in all AML patients in a resource-limited setting. The decision needs to be individualised based on clinical decision implications, and then a high-throughput sequencing method is recommended with variant classification for clinical relevance.
